# Survey of the Relationship Between Metabolic Syndrome and Myocardial Infarction in Hospitals of Urmia University of Medical Sciences

**DOI:** 10.5539/gjhs.v6n7p58

**Published:** 2014-09-18

**Authors:** K. Khademvatan, V. Alinejad, S. Eghtedar, N. Rahbar, N. Agakhani

**Affiliations:** 1 University of Medical Sciences, Urmia, Iran; 2 Center of Reproductive Health Research, Medical Sciences, Urmia, Iran

**Keywords:** incidence, survival, myocardial infarction, metabolic syndrome, Urmia

## Abstract

**Background and Aim:**

The aim of this study was to determine the relationship between metabolic syndrome and myocardial infarction in patients admitted to the hospitals of Urmia University of medical sciences.

**Methods:**

A case-control study population consisted of 172 patients with heart failure who were admitted to Seyedolshohada Hospital. In this method, the researchers present in the units and along with demographic questionnaire of patients, laboratory results needed for the survey (fasting blood glucose, triglycerides and HDL) with waist circumference size, blood pressure, height and weight were examined. Data after collection were analyzed using SPSS statistical software.

**Results:**

In this study of 172 patients with myocardial infarction, 56 patients (38.4%) patients were females and 112 (17.9%) were males. 1.2% of the patients were single, 84.8% were married, 0.6 were divorced and 13.5% were widowed, 116 patients (67.4%) with features of metabolic syndrome and 56 patients (32.6%) were lacking. In this study, females with myocardial infarction had more metabolic syndrome than males and in people whom relatives have a history of heart disease and also people who are overweight as well as obesity and also have features of metabolic syndrome and mean profiles of HDL, LDL, BMI, fasting blood glucose, triglyceride and waist circumference in males compared to males is higher. However, history of smoking, average number of cigarettes used per day, height and weight of males is higher than females.

Other findings indicate a significant relationship between age and sex and having or not having a family history of heart failure, having or not having history of certain drugs and BMI of patient with metabolic syndrome. But a significant relationship was not found between the marital status, education, residence, income, previous history of heart disease, PCI, LDL, history of drug use, type of infarction, the extent of ejection and location with syndrome patients. In terms of survival, because none of the subjects in the study period had expired, this extent was not quantifiable.

**Conclusion:**

Considering the high prevalence of this disorder in Iran and that the high incidence of serious effects imposes on the health care system and that these disorders are somewhat flexible, effort towards lifestyle changes particularly healthy diets, physical activity, weight management and blood pressure, especially in women should be considered.

## 1. Introduction

Cardiovascular diseases include chronic illnesses that are not only considered to account for a high mortality, but also reflect the long term nature of their disabilities and some of the limitations in one’s life (Braunwald, Zipes, & Libby, 2012). According to the World Health Organization in 1996, cardiovascular diseases include about 20 percent of the causes of death in the world. American Heart association estimates that more than 7 million of those diseases, who had experienced myocardial infarction or angina, are still alive and the number of patients with myocardial infarction due to a relapse is readmission in hospitals (Gomez-Marin et al., 1987). Effects of myocardial infarction include: Irritation of the myocardium associated with specific symptoms and signs, physical condition changes, severe mental protests, the loss of job security, loss of recreational activities and social relations, future anxiety and disorders in the relationship between the role of individuals and families. Consequences such as premature fatigue, activity intolerance, fear and anxiety in patients with myocardial infarction cause patients show a new affiliation with the members of the family (Lamas et al., 1997). Definition of myocardial infarction, is ischemia, and cut off blood flow to the heart muscle tissue for more than 20 to 30 seconds that with tissue necrosis makes visceral discomfort in the chest (Braunwald et al., 2012) that causes changes in physical conditions and severe psychological protests that if not lead to death, affected the quality of life of patients and their family members, especially spouses leading to the loss of job security, loss of recreational activities and social relations, future anxiety and disorders in the relationship between the individuals, premature fatigue, activity intolerance, fear and anxiety and affiliation with the members of the family (Billing, 1988). Myocardial infarction occurs mostly in those aged over 40 years, though there may be individuals aged 30 years or even 20 years are also suffered attacks of angina or heart attack (Bagherian, 2008). However, today because of industrial societies and increase palpitate, impaired nutrition, smoking and etc. age of developing heart disease reduced and its incidence is increasing. The mortality rate from the disease in males is far more than females, and women with a delay of about 10 years old will die after men. Statistics show that worldwide, an average of at least 220,000 people within 1 hour after the onset of heart attack symptoms and prior to hospital arrival death due to acute myocardial infarction (Verderose, 2001).

Risk factors that may cause the onset of coronary disease are classified into three categories: non-modifiable, modifiable factors and contributing factors. Old age, male sex, family history of premature atherosclerosis are non-modifiable risk factors, hyperlipidemia, hypertension, diabetes mellitus and smoking are modifiable risk factors and high-fat diet and reduced physical activity are contributing factors in the mortality from myocardial infarction (Zipes et al., 2005). Despite vast improvements in diagnosis and treatment, the disease remains a public health problem in industrialized countries and is the third leading cause of death (Bagherian, 2008). In the developed countries each year from 12 million people 5 million and in the developing countries from 40 million people 10 million lose their lives due to heart disease (Jahanbin, 2009). In Iran, like other industrialized countries, deaths from diseases of the coronary artery is increasing and from 46.1% in 1375 reached to 58% in 1379 and 63.3% in 1385. (Statistical Centre of Iran, 2006) And the leading cause of death in people aged above 35 years (Mohammadi et al., 2006). Today, with the establishment of intensive care units, the mortality of patients with heart failure reduced but the subsequent hospitalization rates are increasing (Verderose, 2001). Cases such as Failure to observe and follow such diet and medication, unplanned discharging and lack of awareness of risk factors and treatment regimens resulted in admission and readmission of patients with myocardial infarction (Dracub, Dunbar, & Barcer, 1995). Which has resulted in enormous costs of re-admissions and according to the World Health Organization medical costs due to frequent hospitalization of patients with coronary artery disease has been estimated about 150 million dollars a year (Ignatavicius, Workman, & Misher, 1999). Reducing re-hospitalizations and improve health in patients with myocardial infarction, require a change in lifestyle that encompasses issues like smoking, non-cholesterol diet, regular exercise and others (Strub, 2002). The studies revealed that patients in employing such a lifestyle, need a number of training, and are lack of sufficient data (Chae, 1999). Search for information by patients with myocardial infarction, reflecting the fact (Lukkarinen & Hentinen, 1997).

Metabolic syndrome is a collection of metabolic abnormalities including abdominal obesity, hyperglycemia, dyslipidemia and hypertension that is associated with overweight and obesity and is also considered as risk factors for cardiovascular disease and diabetes. Metabolic syndrome is associated with overweight and obesity, and is also considered as a risk factor for cardiovascular disease and diabetes. One-third of Iran’s population is infected with the metabolic syndrome that is more prevalent in females (Azizi et al., 2003). Metabolic syndrome is a cluster of metabolic abnormalities, including insulin resistance, hyperinsulinemia, hypertension, dyslipidemia and abdominal obesity, which can lead to cardiovascular disease (Reaven, 1998). Metabolic syndrome is a condition of unknown etiology and numerous clinical protests. Therefore, definition of this syndrome based on the etiology and pathology is not possible and the syndrome is defined by phenotype (International Diabetes Federation, 2005). Metabolic syndrome is a set of risk factors based on insulin resistance associated with hypertension, dyslipidemia, and other metabolic disorders which increases blood pressure and risk of cardiovascular disease, including myocardial infarction (Ozanne & Hales, 2002). Due to adverse effects of metabolic syndrome on cardiovascular diseases, including myocardial infarction, researchers sought to determine the prevalence of this syndrome in patients with myocardial infarction and also comparison of these patients with those without this syndrome have suffered myocardial infarction. The results of this study in adopting the methods of preventing and treating patients, so as to coincide with the treatment of MI for the presence and complications of metabolic syndrome will be effective.

## 2. Methods

In a case-control study, 172 patients with heart failure who were admitted to Seyedolshohada Hospital were studied. In this method the researcher present in the units and laboratory results needed for the survey (fasting blood glucose, triglycerides and HDL) along with waist circumference size, blood pressure, height and weight were examined also the demographic questionnaire was completed. Sample size and data analysis methods: In this study, the following equation was used to determine sample size.


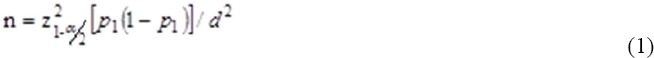


Considering the amount of P1 and previous studies was considered 0.8 respectively. In this study, the value of d was considered 0.1. Considering the level of confidence 0.05 the value of Z was equal to 1.96. In view of the above values and previous studies, sample size was obtained 172 patients. Results obtained using the statistical software SPSS 16 and analyzed with t-test and ANOVA (Sajadi et al., 2013). In the inclusion and exclusion criteria of the study, patients must be referred because of this cause for the first time, lack of other systemic chronic diseases and are able to answer questions and assessment, from Urmia and accurate mailing address and phone number for follow-up and was satisfy for participation in the study.

## 3. Results

In this study of 172 heart patients, 116 (67.4%) patients with features of metabolic syndrome (infected group) and 56 (32.6%) patients were in non-infected group. 56 (38.4%) of the patients were females and 112 (61.6%) were males. 1.2%% of the patients were single, 84.8% were married, 0.6 were divorced and 13.5% were widowed. 10.1% of patients were unemployed, 4.1% were employees, 14.8% were in market and 71% were of other businesses.64.1% of the study population was illiterate, 24.1% of patients were under diploma, 6.5% were diploma, 2.4% were upper-diploma, 2.9% were BA. 28.1% of patients had a history of heart disease. 32.2% of patients had a history of heart disease in their families and 34.4% also had a history of a particular drug. 87.7% of individuals had normal LDL and 65.9% had ST-MI disease. 1.2% of patients had BMI less than 18.5 and 27.5% had BMI between 18.5-24.9, 46.2% had a BMI between 25-29.9 and 25.1% of patients had a BMI greater than 30 (Table 1).

**Table 1:** Distribution of samples according to the BMI index separation group

Variable	Non-metabolic syndrome	Metabolic Syndrome
Age	38/13 ± 83/60	27/13 ± 52/60
Sex		
Male	69 (5/59%)	43 (8/76%)
Female	46 (7/39%)	10 (9/17%)

Special drug use		
Have	40 (5/34%)	26 (4/46%)
Do not have	63 (3/54%)	23 (1/41%)
History of heart disease in families		
Have	44 (9/37%)	11 (6/19%)
Do not have	72 (1/62%)	44 (6/78%)

BMI		
5.18>	0 (0%)	2 (6/3%)
9.24–5.18	24 (7/20%)	23 (1/41%)
9.29–25	55 (4/47%)	24 (9/42%)
30<	37 (9/31%)	6 (7/10%)

According to Table 1 prevalence of metabolic syndrome in males who have had a heart attack the first time was 60% and for women the incidence was 39.7% and the prevalence of metabolic syndrome in patients taking certain drugs was 38.8% while for patients who had no specific drug was 61.2%. The prevalence of metabolic syndrome in patients with a history of coronary heart disease in families is equal to 37.9% and for patients who had no history of heart disease in relatives is equal to 62.1%. Prevalence of metabolic syndrome in patients with a BMI of less than 18.5 was 0% and in patients with a BMI of between 24.9–18.5 was 20.7%, and in patients with a BMI between 25–29.9 was 47.4% also in patients with a BMI greater than 30 was 31.9%. Comparison of the two groups, with regard to profile, LDL, HDL, FBS, BMI, history of smoking years, number of cigarettes smoked per day, number of hospitalizations, triglycerides, low blood pressure, high blood pressure, weight, height, waist circumference are shown in Table 2.

**Table 2:** Mean and SD of profile, LDL, HDL, FBS, BMI, history of smoking years, number of cigarettes smoked per day, number of hospitalizations, triglycerides, low blood pressure, high blood pressure, weight, height, waist circumference of patients in divided group

Variable	Infected	Non-infected	Total
Number	116	56	172
FBS	127.86±59.95	143.95±71.14	132.76±2.28
HDL	40.23±13.47	41.93±9.74	40.78±12.38
LDL	95.29±28.24	98.38±29.25	96.3±28.52
BMI	28.73±7.7	25.68±4.17	24.74±6.89
History of smoking	2.71±2.27	2.86±2.32	2.76±2.28
Number of cigarettes smoked	9.02±14.61	7.87±11.88	8.65±13.76
Number of hospitalizations	1.85±0.07	1.79±0.863	1.83±1.01
Triglycerides	167.02±104.2	119.66±114.07	151.6±109.47
High blood pressure	129.03±24.16	124.57±25.72	127.58±24.7
Low blood pressure	82.26±15.78	79.73±16.2	81.44±15.91
Weight	76.07±13.79	70.61±11.87	74.29±13.41
Height	163.59±11.92	165.96±8.9	164.36±11.06

Waist circumference

P <0.001 compared with men * the difference is significant.

According to the results in Table 2 average BMI, triglycerides, waist circumference, and weight in infected group compared to non-infected group is higher. The differences in the above cases show significant differences between the groups (Table 2).

## 4. Discussion

Given the output of the logistic regression model, age, gender, family history of heart failure, certain drugs, and BMI of patients were effective in diagnosis of metabolic syndrome features. As a result, patients with features of increasing age, being female, and BMI over 25, having a family history of heart disease, certain drugs have more chance to have the characteristics of metabolic syndrome than others. Average BMI, triglycerides, waist circumference, and body weight were higher in the infected group compared with the control group. According to the results of this study, females had more metabolic syndrome than males. Based on research conducted in Mashhad 40% of people had metabolic syndrome, and these figures are about the same in other cities while the prevalence of metabolic syndrome in America 25 percent and in urban areas such as Nancy, France, is less than 10 percent. Prevalence of metabolic syndrome in females of Mashhad is estimated 55% and in males 25% while in most countries its prevalence in females is much less than males. Improper food habits, rural to urban migration and reduce the amount of physical activity are considered important factors affecting the increased prevalence of metabolic syndrome in Iran and said: Unfortunately, a change in eating habits is the most important reason is the increased prevalence of these diseases (Mashhad.irib.ir). People who have a family history of heart disease and obesity, as well as those who are overweight have more features of metabolic syndrome. Average profiles of HDL, LDL, BMI, fasting blood glucose, triglyceride levels and waist circumference in females compared to males is higher. However, history of smoking, average number of cigarettes used per day, height and weight of males is higher than females. In a study conducted on a population of Iran prevalence of metabolic syndrome in females was reported more than in males (Azizi et al., 2003), which was confirmed by our study. In an extensive case study that was conducted simultaneously in 52 countries, it was found that the relationship between metabolic syndromes with MI is equiponderant with diabetes mellitus and hypertension and was operate significantly stronger than the other risk factors (Mente et al., 2010).

In a similar study conducted in Croatia, it is concluded that the consequences of MI statistically have not significant difference between the groups with or without metabolic syndrome. Even the amount of LVEF between the two groups was similar, which was aligned with the results of our study group (Zdravko et al., 2011). In a study conducted in Nepal prevalence of metabolic syndrome in patients with myocardial infarction was reported 26.19% which is very different with the result of this study that is 76.4%. Perhaps the relationship between genetic structure of Nepalese men and unimportant role of inheritance in making up coronary disease and MI in that land, in contrast to our study, heredity plays an important and significant role (Pandy et al., 2009). In a study on patients with acute myocardial infarction less than 45 years revealed that among 165 young patients with MI about 3.2% had metabolic syndrome. Indicating the role of syndrome in making atherosclerosis and infarction and by the prevalence of metabolic syndrome in our study that was 67.4% was quite similar, and it may be concluded that the occurrence of MI is in patients in younger age groups (Stuart et al., 2006). In the current study, the significant relationship between sex and having or not having a family history of heart failure, having or not having certain drugs and BMI are of effective factors in detecting between the two infected and non-infected groups but between marital status, jobs, education, residence, income, previous history of heart disease, PCI, LDL, history of drug use, type of infarction, the extent of ejection and location of infarction, there was no significant relationship (P> 0.05). In a research was reported that metabolic syndrome is a significant cause of myocardial infarction (Ninomiya et al., 2004). In another study, LDL level in patients with coronary heart attack was 2.2 times more than healthy people (Alexander et al., 2003). In a study also found that metabolic syndrome doubles the risk of heart attack and this risk is in males 1.5 times more than in females (Grundy et al., 2004), while the current study did not show a relationship.

In the next study, high triglyceride was the most important criterion and then blood sugar and high blood pressure had that role (Gustafsson et al., 1998). In our study, the significant association of triglycerides, weight, waist circumference and BMI were shown. A research that was took on ten thousand patients about the relationship between metabolic syndrome and stroke and heart attack it was identified that between this syndromes and the two cases mentioned, there is a significant relationship (Ninomiya et al., 2004). It was also identified that likely to develop the syndrome increases with age in developing countries and 40 percent of people over forty years suffered this problem and there is no difference between females and males in the disease (Ford et al., 2002). This disease increases the risk of death from heart disease with incidence of stroke (Haffner et al., 1992; Girman et al., 2004). In another study, for more than 700 women with heart disease and metabolic syndrome was diagnosed higher mortality than women without metabolic syndrome (Marroquin et al., 2004). A research also revealed that at least three components are sufficient to confirm the metabolic syndrome but the presence of even one of these components would also create the incremental risk of the rest of cases (Malik et al., 2004). The research revealed that the risk ratio for heart disease in females than in males is 2.58 to 1.74 (McNeill et al., 2002). Research in Finland on 1209 patients without stroke, diabetes and cancer showed those people were not likely to have characteristics of patients with metabolic syndrome (Lakka et al., 2002). The research also concluded that cardiovascular disease and mortality from it in men and in people with metabolic syndrome even in those who have not been diagnosed with diabetes has increased. Early identification, treatment and prevention of the disease and deal with the epidemic of sedentary lifestyle and weight gain are a major challenge in the treatment group (Groop & Orho-Melander, 2001). Finally, research suggests that metabolic syndrome is significantly associated with a history of stroke and heart attack and also concluded that cardiovascular disease and mortality from it in men and in people with metabolic syndrome even in those who have not been diagnosed with diabetes has increased. This study introduced the clinical significance of the metabolic syndrome as a risk factor for cardiovascular disease and proposed the need to adopt strategies to control the disease and diseases caused by it. Early identification, treatment and prevention of the disease and deal with the epidemic of sedentary lifestyle and weight gain can be a major challenge in the treatment group (Deedwania, 2004). In terms of survival, because none of the subjects in the study period had expired these levels were not quantifiable. In a study to assess infraction viability in patients with metabolic syndrome, syndrome, 494 infected cases and 469 non-infected cases were studied and was identified that having or not having metabolic syndrome have not a marked influence on the mortality rate of patients with myocardial infarction (Won et al., 2013).

## 5. Conclusions

In terms of survival, because none of the subjects in the study period had expired these levels were not quantifiable that is recommended for the long run of such research. Given the high prevalence of this disorder in Iran and that imposes a high incidence of serious effects on the health care system and because these disorders are somewhat flexible and will offer training programs for changing the lifestyle specially education for healthy diet, physical activity, weight control and blood pressure be taken into account seriously.
